# Engaging Black men who have sex with men (MSM) in the South in identifying strategies to increase PrEP uptake

**DOI:** 10.1186/s12913-022-08914-2

**Published:** 2022-12-07

**Authors:** Latrice C. Pichon, Michelle Teti, Shanell McGoy, Velma McBride Murry, Paul D. Juarez

**Affiliations:** 1 School of Public Health, Division of Social and Behavioral Sciences, The University of Memphis, 3825 Desoto Ave 209 Robison Hall, Memphis, TN 38152 USA; 2 Department of Public Health, The University of Missouri, 806 Lewis Hall, Columbia, MO 65211 USA; 3 Formerly Tennessee Department of Health, 710 James Robertson Pkwy, Nashville, TN 37243 USA; 4 Departments of Health Policy Human and Organizational Development, Vanderbilt University Medical Center & Peabody College, PMB 90 230 Appleton Place, Nashville, TN 37203 USA; 5 Department of Family & Community Medicine, Meharry Medical College, 1005 Dr. D.B. Todd Jr. Boulevard, Nashville, TN 37208 USA

**Keywords:** HIV, Pre-exposure prophylaxis (PrEP), Young Black/African American men who have sex with men (MSM)

## Abstract

**Background:**

Promotion, uptake, and adherence of pre-exposure prophylaxis (PrEP) is paramount to ending the HIV epidemic among young Black men who have sex with men in the South. The purpose of this study was to explore strategies needed for and barriers to PrEP uptake needed to achieve HIV prevention goals identified in the U.S. Department of Health & Human Services initiative to reduce new HIV infections in the United States by 90 percent by 2030.

**Method:**

Young adults (*n* = 25) between the ages of 15–34 were recruited from community-based organizations in Memphis to participate in four focus group discussions. Discussion topics included motivations, barriers, and facilitators to PrEP use. Data were analyzed using thematic analysis.

**Results:**

All (100%) of participants self-identified as HIV-negative, Black (96%), men who have sex with men (96%), and currently prescribed PrEP/Truvada (60%). Themes identified for increasing uptake included 1) trusted peers, 2) relatable healthcare provider (e.g., Historically Black College and University (HBCU) trained, LGBTQ), and 3) use of social media. Mislabeling of PrEP as promiscuity promoting and limitations with PrEP marketing (e.g., solely LGBTQ) were recognized as barriers that perpetuated stigma.

**Conclusion:**

Findings suggest the importance of increasing awareness among health professions students matriculating at HBCUs of their perceived role as relatable healthcare providers by Black MSM; working closely with couples; and crafting of PrEP messaging that is non-stigmatizing. Findings will inform public health interventions for young Black MSM and facilitate HIV prevention efforts with other groups disproportionally affected by HIV in the South.

## Background

In the United States (U.S.), new HIV infections are disproportionally high among young, Black, adult men who have sex with men (MSM). In 2019, 9,421 of the 36,801 (26%) of new HIV infections were among Black MSM [1]. When accounting for age, 3 of 4 Black MSM (who were identified as HIV +) fell within age range of 13–34 [1]. The prevalence of HIV and poor HIV health outcomes is greatest in the U.S. South. While just over a third of the U.S. population lives in the South, more than 50% of HIV incidence is reported from this region [2]. A critical objective of the updated National HIV/AIDS Strategy (NHAS) is reducing HIV disparities in the South [3]. A more recent initiative, Ending the HIV Epidemic (EHE): A Plan for America, found that 48 jurisdictions accounted for more than half of new HIV diagnoses [4]. In 2018, Memphis (site of the current work) ranked fourth among metropolitan statistical areas for incidence of HIV with a rate of 530.3 per 100,000 [5]. There were 308 total new diagnoses in Memphis/Shelby County in 2018 [6]. Of those, young adult Black MSM between the ages of 18–34 accounted for 57% of the new infections in Memphis [6].

PrEP is a daily pill that has been found to prevent HIV transmission by 92% [7]. PrEP was approved for consumption in early 2012 by the Federal Drug Administration (FDA) [8]. Increasing access to HIV prevention services and the promotion, uptake, and adherence of pre-exposure prophylaxis (PrEP) is paramount to ending the epidemic of HIV, in general, and among young, adult, Black MSM in the South, in particular. In 2017, data from 23 U.S. cities found that 78% of Black MSM were aware of PrEP, however, only 19% of Black MSM reported using it [9]. Uptake among Black MSM living in the Southern region of the U.S. lags behind other regions of the country partly due to a lack of a well-defined continuum of care [10, 11]. A survey conducted in 2017 that included 724 PrEP eligible clients participating in a Memphis-based PrEP demonstration project, found that only 17.1% were prescribed PrEP [12].

Black MSM living in the South encounter a number of challenges in accessing evidence-based HIV prevention services, including PrEP. Low income and predominantly racial and ethnic minority people who live in the South are at disproportionate risk for HIV transmission, including Black MSM, due to social determinants of health, such as lack of transportation and insurance, but also due to longstanding social injustices imposed on Blacks in the South, that are vestiges of slavery and Jim Crow Laws [13]. There also are fewer medical providers that prescribe PrEP in predominantly low income, Black neighborhoods [14]. Black patients also report infrequent requests for their input about treatment decisions, and experience less evidence of patient-centered care by health care providers [15]. In 2015, 34% of transgender women reported having at least one negative experience related to being transgender when seeing a health care provider [16]. Historical subjugation of young, Black MSM to economic, social, and political disadvantages supported by structural racism, implicit biases, and related conditions work in tandem with other social determinants to dissuade help-seeking behaviors among young Black MSM [13].

The ways in which health behaviors are influenced and affected by structural oppression suggests a need for greater consideration of how social determinants of health present barriers to PrEP uptake among young adult Black MSM in the South. Understanding the effects of macro, socio-economic-political structures on individual-level, HIV preventive behaviors and the factors that contribute to low PrEP uptake, inequities and disparities in HIV care, among young Black MSM provides a promising avenue for research. Increased understanding of how contextual factors such as conservative beliefs, sexuality-related discrimination, stigma, and lack of PrEP knowledge among medical providers contribute to low access by young black MSM is sorely needed [17]. Additional information about the role of social barriers including stigma related to PrEP use, self-identifying as gay, or being Black along with normalizing discussions about HIV prevention and sexual health also is needed [18, 19]. To address these barriers, we sought to identify strategies and barriers to increase PrEP uptake among Black MSM young adults.

## Methods

### Setting

Memphis, TN is 1 of 48 U.S. jurisdictions that account for more than half of new HIV diagnoses. It is an urban city in Tennessee located in the tri-state area bordering rural parts of Northern Mississippi and Eastern Arkansas. During the time of the project, Memphis had recently started PrEP navigation at four community-based organizations and the local health department [20]. Memphis, like other Southern cities is located in the Bible belt which has a history of strong religious influence on daily life, shaping beliefs associated with sexuality, discrimination, and HIV-related stigma [21, 22].

### Recruitment

The first author worked closely with PrEP navigators–persons hired at four, community-based organizations, the local health department, and other trusted members of the community to identify and recruit persons who could benefit from PrEP to participate in focus groups. Recruitment flyers and project information were developed in collaboration with a LGBTQ + advisory board and an HIV coalition of faith- and community gatekeepers. Eligibility criteria included: persons self-identifying as: 1) Black or Latino, 2) MSM or transgender female, and 3) aged 15–34 years. The final sample included 25 persons, an appropriate and sufficient sample size for qualitative research [23]. Two University Institutional Review Boards approved this research [University of Memphis Protocol # PRO-FY2017-528 and Meharry Medical College #16–08-593].

### Procedures

A total of four, focus groups (average size *N* = 6) were conducted between August 2017 and October 2017; two were comprised of current PrEP users and two were comprised of non-PrEP users [23]. The first author has expertise in qualitative methods and facilitated focus groups. She was assisted by a note-taker. Focus groups used a semi-structured interview guide exploring barriers and facilitators of PrEP use (see Table 1). The facilitator probed for history and context of PrEP experiences and barriers at individual, relational, and social levels. Prior to the start of the focus group, participants were provided the informed consent document to read along with the focus group moderator which provided an opportunity to ask the moderator questions. Following questions and clarifications, participants proceeded with verbally consenting to participate in the discussion (as approved by the ethics committee). Each discussion lasted approximately 1.5–2 h. One focus group was held in participant’s home at a convenient time and location and three were held at LGBTQ + community agencies. Participants received a $50 Visa gift card for their time and complimentary refreshments.

**Table 1:** Focus group discussion guide

Domain	Questions
Motivations	-What would motivate you to take PrEP daily? -Who would motivate you to take PrEP daily? -What are your most important needs?
Barriers	-What are your concerns about PrEP medication side effects? -What barriers will prevent you from taking PrEP?
Facilitators	-What are your thoughts about the effectiveness of PrEP in reducing HIV risk? -What are effective strategies to increase PrEP awareness?
PrEP Delivery	-What health or social services do you use? -What is your most important health concern about taking PrEP daily? -What suggestions do you have for improving linkage to PrEP services?
PrEP and Sexual Decision Making	-What would encourage consistent condom use while taking PrEP daily?

### Analysis

All focus groups were audio-recorded and transcribed verbatim. The first author verified transcripts and removed any identifying information. Data were managed with Atlas.ti and analyzed using thematic analysis to capture the key patterns in the way that the participants talked about PrEP [24]. Analysis steps included initial and more specific coding, analytical memos, and organizational matrices and reports. The first and second authors (i.e., the coders) reviewed the transcripts to conduct initial coding of the data. The goal of the first round of coding was to identify all possible patterns in the data related to PrEP strategies and barriers informed by the social determinants of health. Coders assigned descriptive code labels to common words, phrases, and topics. Coders reviewed the data separately and then met monthly for 1 year to discuss and compare their initial descriptive lists.

Next, the coders reviewed the data in more detail. Preliminary codes were sorted, collapsed or expanded into three themes of facilitators and three themes of barriers by identifying similarities and differences between codes. Coders created a codebook that identified and defined the themes, and then using the codebook, matched interview text excerpts to codes, and met weekly until sufficient coder agreement and saturation was reached. Coders created a matrix that outlined and defined each of the final themes and a report that listed example quotes under each theme, to organize the data for the results section. The first author, who facilitated discussions, reviewed the matrix and report to ensure that the data appropriately reflected the content of the interviews to maintain rigor [25].

## Results

There was a total of twenty-five, focus group participants. All participants self-identified as HIV-negative (100%). Fifteen participants were currently prescribed PrEP (60%). We conducted four focus groups. The composition of each group had observable differences by age and PrEP status. In 2 of the 4 focus groups, participants ranged in age from 18–25 years. The remaining two focus groups were older young adults between the ages of 26–34. The two younger adult discussion groups were composed of PrEP users. The sample comprised of mostly Black (96%) men who have sex with men (96%). While recruitment efforts and eligibility were inclusive of other LGBTQ + and minority groups, we were only able to reach 1 Black transwoman and 1 Latinx cisgender male. Finally, in 2 of the 4 groups two couples attended the discussion together. In general, there were few differences in responses among participants taking PrEP compared to those not taking PrEP. For example, PrEP users had personal experience about adverse effects of the medication and were able to share how these symptoms were temporary and subsided. However, despite PrEP users receiving education about PrEP, there were still some gaps in knowledge and misinformation across all groups no matter PrEP status.

Emerging themes identified by participants to increase PrEP uptake included the importance of relationship status, trusted and relatable sources of healthcare (e.g., Historically Black College and University (HBCU) trained and LGBTQ +), and use of social media for PrEP messaging (see Table 2). Addressing PrEP-related stigma, misuse of PrEP as promiscuity promoting, and limitations with PrEP marketing (e.g., solely LGBTQ +) were recognized as barriers to uptake and perpetuating stigma (see Table 3).

**Table 2:** Facilitators of PrEP uptake

Theme	Representative Quotation
Relationship Status	*I was talking to somebody that had HIV and I really liked him, so that’s what encouraged me to get on PrEP even quicker.* FG2
	*If you’re in a relationship, you won’t want to use a condom if both of y’all on PrEP. We don’t use a condom, we trust each other, we on PrEP, we’re good.* FG2
	*When I’m in a relationship I don’t use protection, is one reason.* FG3
Trusted and Relatable Source	*People within the field, health providers, your outreach specialist, if they are on it [PrEP]. If they say ‘I’m speaking because this is what I’m on.’* FG4
	*Somebody who a lot of people look up to in this community…Reach out to people who got a lot of people looking at them already.* FG3
	*Somebody known to advocate for it [PrEP], then more people would be like, ‘Oh, this is somebody I look up to or know,’ they have more credibility.* FG2
Messaging	*Once PrEP becomes for everybody, gay men who probably need it, will become participants more.* FG1
	*Broadening so doesn’t look like it’s targeted just for one group.* FG4

**Table 3:** Barriers of PrEP uptake

Theme	Representative Quotation
Marketing ‘gay pill’	*In [city name omitted], I don’t think I’ve heard much at all about PrEP. Even going to places like [name of agency removed] and getting tested, nothing immediately stood out, that this is talking about PrEP….. I feel like HIV here in the South is still stigmatized in a way, that I didn't feel back at home.* FG1
	*PrEP is marketed toward gay men purposefully…. But, I don’t see that marketing that goes toward heterosexuals who are still affected by HIV. It’s still looked at as a very gay disease.* FG1
Promiscuity Promoting	*I know a few select people that are on PrEP, I think another barrier that has developed for me is you use PrEP to have unprotected sex versus it being used as additional protection. It’s one of those things, I’m on it. I can just have raw sex any time all the time.* FG4
	*If I know that I can take this pill and not catch HIV, I think everyone will be a little more loose and not use condoms…. That is an issue in the back of my mind, people not using condoms as a result of taking the pill.* FG1
	*Because they’re on PrEP, they think they can sleep around all the time unprotected. ‘Just because I'm doing this, I’m protected. I can have multiple sex partners, I can sleep around without using protection.’* FG3
PrEP Access	*White men particularly are the face of the community and taking PrEP…. our communities just are not exposed to for whatever reasons that our white counterparts have the advantage of having that information more readily available to them, particularly here in the South.* FG1
Linkage Process	*Some of the requirements to get on PrEP are crazy. You have to say you slept with so many people.* FG2
	*Process is taking so long. My doctor didn’t know what it was and started trying to get that in motion [sigh].* FG4

### Facilitators of PrEP uptake

#### Relationship status

Participants expressed varied perspectives of involvement of their primary sexual partner in their decision to use PrEP. In the two focus groups with current PrEP users, couples discussed how uptake occurred because one partner started using PrEP and encouraged the other to do so too. Discordant serostatus also served as a facilitator for uptake for several couples. Other factors that facilitated PrEP uptake included participants were more sexually active, had multiple sexual partners, or were in a serious committed relationship where condoms were not being used. One participant explained:

Going into a relationship where it’s a serious, committed relationship, I know condoms would be thrown out the window. With PrEP, I am protecting myself and my partner. That's probably the only precaution of why I would even consider PrEP. I’m not in a relationship phase at this moment. FG4

Other participants across focus groups explained the uncertainty of ‘extra-curricular’ sexual activities their partner was engaging in and considered PrEP as an option to protect themselves from acquiring HIV. In sum, features of relationships often prompted discussions and use of PrEP.

#### Trusted and relatable source

In the discussions with non-PrEP participants, the importance of characteristics of the physician prescribing PrEP were discussed at great length. Participants said that the physician should be relatable, trusted, and knowledgeable about PrEP. Some defined “relatable” as having a medically trained MSM physician who can converse with the patient. One participant explained:

When I was with a gay physician it was like, ‘Yes, I did this. Should I have done that?’ I feel a lot more comfortable and transparent in having those dialogues. FG1

Other participants stressed the importance of the physician having received training at a Historically Black College/University.

Someone from Meharry, someone from Howard. I’m familiar with how doctors are trained at HBCU medical schools. That’s something that I trust. My better treatment has come from people in those spaces, and I've heard better stories about doctors trained from those spaces, because they look at things differently. Because everybody in there is Black. FG1

Another trusted source facilitating PrEP uptake was members of social and sexual networks. Participants discussed the importance of a peer influencer that appealed to younger age groups and a person respected by members of the LGBTQ + community such as a trans adviser, gay families, drag families, and /or J-setter dance teams. Being relatable to someone from the Black LGBTQ + community that is currently on PrEP offered credibility and motivation for PrEP uptake. One participant said:

It’s relatability. To have somebody within the community that’s speaking to it. Probably on the actual medication. FG4

#### Messaging

While all participants self-reported basic knowledge about PrEP, questions remained across all four groups along with a desire for additional information. There was the belief that more information about PrEP should be disseminated on social media outlets (e.g., Facebook, Instagram, Pornhub, Snapchat) and hook-up apps to increase uptake and to promote adherence among those currently on PrEP. The language used on Hook-up apps include examples like, “Oh, I’m on PrEP” and, “let’s do this, I’m on PrEP” and visibility was viewed by participants as a mechanism to facilitate some aspects of normalizing the larger discussion of PrEP as a preventative tool.

Recommendations were made to destigmatize PrEP via social media and use sex positive messaging tailored to broader audiences including cisgender men and cisgender women of all ages. One participant from FG4 suggested “make it accessible to everybody”. Another participant questioned: “Why are you targeting the younger age men? What about the older people?”. The participants wanted messaging to reach everybody and to promote PrEP education at local events attended by a broader audience (i.e., 5Ks, job fairs, street festivals, nightclub/lounge). These factors were thought to be facilitating factors to increase uptake.

### Barriers of PrEP uptake

#### Marketing PrEP-related stigma

Participants gave examples of several limitations with PrEP marketing. Most felt PrEP was geared exclusively to gay men as one participant said: “It's a gay pill.” This was problematic for participants living in the South as one participant noted:

Here [city name removed] being gay in general is still stigmatized, which means that the risk of HIV is definitely stigmatized. Any type of preventative measure is still an admittance that you are a participant, which is definitely very, very taboo. FG1

Participants collectively noted other higher risk heterosexuals and injection drug users may equally benefit from PrEP. They believed that limited marketing of promotion of PrEP solely to MSM was a barrier for uptake among young people who may not identify as a sexual gender minority, for those undergoing identity development, and those still shaping their identity. One participant shared:

You’ve had multiple sexual partners before you can truly stand in your truth with your sexuality, and at that point, how many risky encounters have you had? FG1

#### Promiscuity promoting

Participants raised concerns with stigmatizing messaging of the mislabeling of PrEP as promiscuity promoting. They said that PrEP uptake was slowed by community attitudes around the drug being mis-used as a means to engage in reckless condom-less raw sex acts with multiple sex partners. Participants said that they were reluctant to get on PrEP in fear of being labeled a “Truvada whore”, the perception of individual’s permission to have multiple sexual partners.

#### PrEP access and linkage process

Accessing PrEP services and information was also stigmatizing and discriminatory to participants. Discussants spoke of stigma in the context of PrEP clinics being affiliated with HIV care. One of the known PrEP providers also treats MSM and transgender women for HIV and going to this facility was a major concern for participants. Youth do not want to be ‘outed’ for being seen at a known healthcare provider for HIV. One participant shared:

I hear people, even my mother, reference she has a friend that suffers from HIV, and is treated at [clinic] so when I told her I was going to [clinic], I immediately got a crazy look. FG3

Participants discussed lack of knowledge and access to PrEP information living in the South. One participant believed White men have more information compared to Black men. There were noticeable differences to information access on social media dating apps as one participant explained the missing drop-down feature for PrEP disclosure on Black apps:

Jack’d doesn't have this [drop-down menu], and maybe just being in the city of [removed] but Jack’d is geared towards African Americans who use that app and Grindr is more geared towards our counterparts. For them to have a drop-down, they get the benefits of the app and we don’t. To me, that’s a problem in itself. FG4

## Discussion

Our focus group discussions with young adult Black MSM highlighted solutions to reduce PrEP uptake barriers and increase strategies to improve PrEP uptake among a population subject to social vulnerabilities. First, data from the current study demonstrates the need for extending PrEP awareness and training to relatable medical providers matriculating at HBCUs, persons with lived experience, particularly from the Black, LGBTQ + community, and current PrEP users in the South. It is essential to have well trained and culturally sensitive providers with similar lived experiences facilitate PrEP linkage. In 2018, the highest prevalence of individuals living with HIV was mostly found in southern states, despite an increase in interest in PrEP use [10]. This likely is related to poor quality care, lack of access, and medical mistrust [26]. The need for trusted and relatable healthcare providers was an emerging theme in the current study which corroborates previous research [19, 27]. A study previously conducted in the Southeast found the sexual identity of the health care provider as a factor in patient comfort. Participants desired better access to LBGT-friendly PrEP facilities, and male healthcare providers who self-identify as gay as well as concordance on Black race [28]. One study found culturally competent primary care providers in HIV care settings delivered more equitable care and had better patient outcomes compared to other providers with lower self-reported cultural competence [29]. Health outcomes among marginalized groups deeply rooted in social and economic disparities could be improved by offering equitable quality health care by patient-centered relatable providers and with proper investment in medical education training programs.

Second, working closely with couples in serodiscordant relationships and anyone engaging in condomless sex practices emerged as an important facilitator of PrEP uptake. This finding is similar to that of Arnold and colleagues (2017) who interviewed young MSM in the Deep South and found social factors such as partners’ serostatus and new sexual relationships as well as behavioral risk factors including anonymous, online, and multiple sex partners as influential factors in PrEP uptake [30].

Third, patient-centered approaches to build rapport with clients may facilitate disclosure of intimate details of sexual practices as important indicators for prescribing PrEP. Towards this end, clinics and community agencies may want to introduce PrEP navigation to current staff to enhance workforce development skills with linking eligible clients to prevention services [20]. Navigation can be used as a strategy to dispel myths regarding medication use identified by our focus group participants and improve barriers to PrEP information and access for those who could potentially benefit for PrEP.

Fourth, attitudes and beliefs about PrEP from current study participants highlight barriers to access which dovetails another previous study which found PrEP-related stigma may impede access to prevention modalities like PrEP among MSM [31]. Addressing the discriminatory practices and bias in healthcare settings is conceivably achievable if evidence-informed approaches like navigation become the standard of care. Finally, the marketing of PrEP emerged in the current study as stigmatizing gay Black men. These findings are congruent with other previous studies demonstrating the importance of addressing PrEP-related stigma as a barrier to learning about PrEP and eventual uptake [18, 19, 32]. Participants in the current study suggested that there is not enough public marketing that prioritizes a broad audience beyond gay men. Eaton and colleagues explain HIV stigma and gender bias towards a subgroup of people living with HIV may be inadequately contributing to translation of biomedical advancements to community uptake [33]. Framing PrEP as a tool only for Black MSM, subsequently perpetuates stigma [34]. Participants in the current study were reluctant to use PrEP for fear of being mislabeled. A study conducted with Black gay and bisexual men from the Mid-west found PrEP use was perceived by participants as an automatic marker of being gay [35]. Therefore, crafting PrEP messaging for broader audiences as suggested by current focus group participants to increase PrEP uptake and destigmatize PrEP is recommended [36, 37]. Additional studies exploring the effects of intersectional stigma on PrEP use among Black MSM is needed to inform health communication strategies. Proper messaging is key to the success of PrEP uptake among systemically oppressed racialized communities to improve access to health information.

## Limitations

The focus on PrEP uptake among participants identifying mostly as Black men who have sex with men limited a more focused discussion on transgender issues. Separate focus groups exclusive to transgender women were beyond the scope of the current study. The timeline these data were collected is another potential limitation as recent data suggests PrEP use is less associated with sexual promiscuity than previous research.

## Implications

Data from the current study have implications for strategies to reduce regional and racial disparities in PrEP uptake as well as strategies to improve health equity for PrEP access at multiple levels of influence. At the individual level, tailored information for all subsets of the population regardless of sexuality should be disseminated via multiple media platforms. At the interpersonal or relational level, evidence based behavioral interventions can be adapted to facilitate conversation with partners. Federal agencies should consider funding PrEP clinical trials, microbicide research, and other formularies as more options for PrEP uptake at HBCUs located in the South to address needed institutional changes. Finally, the utility of trusted Black medical institutions, providers, and researchers conducting research and practice in the southern region are opportunities to better understand the unique needs of patient populations accessing prevention services to address structural challenges of discrimination and racial and gender bias.

## Conclusions

Study findings can inform the development of public health interventions prioritizing Black MSM young adults and help facilitate HIV prevention efforts to other groups disproportionally affected by HIV in the South. Moreover, these focus group findings support the need for community-academic research partnerships to inform strategies used by state and local health departments, federally qualified community health centers, and community-based organizations to increase PrEP uptake.

## Data Availability

The qualitative data generated and analyzed during the current study are not publicly available due to our lack of non-data sharing agreement during the acquisition of data but are available from the corresponding author on reasonable request.

## Abbreviations

EHE: Ending the HIV Epidemic
HBCU: Historically Black College and University
HIV: Human Immunodeficiency Virus
LGBTQ +: Lesbian, gay, bisexual, transgender and queer or questioning
MSM: Men who have sex with men
NHAS: National HIV/AIDS Strategy
PCP: Primary care provider
PrEP: Pre-exposure prophylaxis
US: United States
